# Pseudo-feeders as a red flag for impending or ongoing severe brain damage in Vein of Galen aneurysmal malformation

**DOI:** 10.3389/fped.2022.1066114

**Published:** 2022-12-08

**Authors:** Guillaume Saliou, Silvia Buratti

**Affiliations:** ^1^ Department of Neuroradiology, Centre Hospitalier Universitaire Vaudois (CHUV), Lausanne, Switzerland; ^2^ Faculté de Biologie et de Médecine UNIL, Lausanne, Suisse; ^3^ Dipartimento di Emergenza Ist, U.O.C. Terapia Intensiva Neonatale e Pediatrica, Genova, Italy

**Keywords:** vein of galen aneurysmal malformation, outcome, prognostic factor, encephalomalacia, pseudo-feeders

## Introduction

Many neuroradiological and hemodynamic prognostic factors have been studied in newborns and infants with Vein of Galen aneurysmal malformation (VGAM), aiming to define the best therapeutic management. Severe presentation of VGAM, with high-output congestive heart failure, poor neurological status, and multiorgan failure, is known to be associated with adverse clinical outcomes and mortality. However, in the absence of established brain damage or signs of heart failure at birth, how can we try to predict both the short-term disease progression and outcome?

## Relevance of the subject

**Figure 1:**
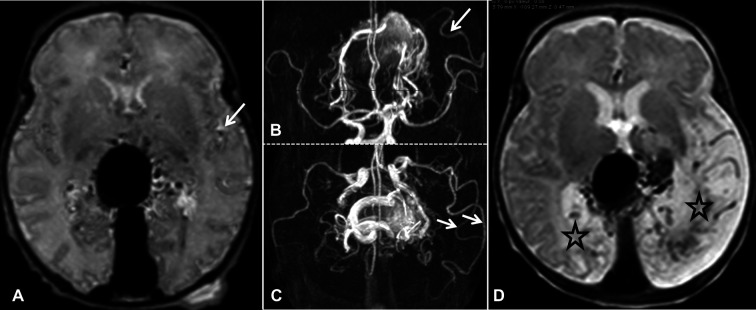
MRI at birth in axial T2-weighted SE (**A**) and TOF in axial and coronal reformat (**B,C**, respectively). The brain parenchyma is Normal. However, abnormal dilations of the distal branch of the left MCA are identified (**B,C**, indicated by arrows). These dilated arteries are also visible in the Sylvian fissure on T2 SE (**A**, indicated by arrow) and correspond to so-called “pseudo-feeders”. Follow-up SE T2 axial MRI performed 19 days after birth (**D**). We observe bilateral encephalomalacia, especially in the brain parenchyma, downstream of the dilated pseudo-feeders in the left parieto-occipital lobe (**D**, indicated by stars).

To emphasize this key point in the management of VGAM, we share an illustrative case, see Figure 1. A baby girl with a prenatal diagnosis of VGAM was born at full term, showing mild signs of cardiac overload and pulmonary hypertension, both well tolerated, and without the need for medication. MRI performed at birth showed normal brain parenchyma (Figure 1A). She was able to bottle feed successfully and was discharged. She presented for follow-up less than three weeks later and was still asymptomatic with a normal neurological exam. Unfortunately, the follow-up MRI indicated extensive brain damage (Figure 1D) and, at that point, endovascular treatment seemed futile. Medical management consisted of conservative care and clinical follow-up.

## Discussion

In our view, this case illustrates three essential points to contemplate in newborns presenting with VGAM. Firstly, cerebral damage from arteriovenous shunts may rapidly evolve in the early stages. Secondly, despite extensive brain damage, a newborn's clinical exam may be normal since the correlation between neuroimaging and clinical status is often unreliable at this age. Therefore, MRI is an essential tool for evaluating the disease progression. Thirdly, it is vital to identify the appropriate time window for treatment, balancing the risks and benefits of early embolization to prevent permanent brain damage.

Are there findings that may help guide the difficult therapeutic pathway in babies with well-tolerated VGAM? We believe the answer is yes, by paying attention to *pseudo-feeders*. Pseudo-feeders are abnormal, dilated branches of the M2 or M3 segments of the middle cerebral artery (MCA) in the Sylvian fissure that are not part of the choroidal system and that should not be considered as part of the VGAM itself. Indeed, these dilated arteries supply the normal brain and not the malformation. They have been previously described as a sign of severe vascular steal phenomena through the shunt, with venous loco-regional hypertension from both the shunt and right-side cardiac failure observed in postnatal (1) and prenatal (2) MRIs. Pseudo-feeders have been associated with a high risk of further brain damage. In the latter publication, MCA pseudo-feeders were identified on MRIs in 53% of fetuses. In the absence of obvious fetal brain damage, they were associated with poor brain outcomes at birth in approximately half of the newborns (*p* = 0.003). Among the 17 patients with only pseudo-feeders visible on fetal MRIs and with no encephalomalacia, nine went on to develop encephalomalacia by the time of birth, over a mean time interval of 34 ± 18.6 days. Moreover, in prenatal presentation, when the fetal pseudo-feeders were unilateral, the encephalomalacia was always located on the same cerebral hemisphere and in the same vascular territory. In our illustrative postnatal case, while encephalomalacia was mainly located in the vascular territory of the pseudo-feeders, limited contralateral encephalomalacia was also visible. In fetuses with no pseudo-feeders visible on the MRIs, no brain damage was observed at birth. Finally, the presence of fetal pseudo-feeders on MRIs was associated with an elevated risk of severe heart failure at birth (*p* = 0.002) as well as severe pulmonary hypertension (*p* = 0.002).

This neuroradiological sign should therefore be treated as indicative of poor hemodynamics in fetuses and newborns. We do not believe that pseudo-feeders reflect vascular steal alone, but rather a combination of high-flow arteriovenous (AV) shunt, venous hypertension, and arterial steal, which, together, lead to progressive parenchymal damage and poor clinical outcomes. This pathophysiological setting is highly related to the systemic hemodynamic consequences of a low-resistance, high-flow cerebral lesion. The relevant venous return, causing dilation of the right heart chambers and pulmonary overflow, as well as the restrictive physiology of the left ventricle, with flow reversal in the aorta and right-to-left shunt through the foramen ovale and ductus arteriosus, account for the negative effects on cerebral blood flow and metabolism.

In conclusion, pseudo-feeders identified on MRIs obtained at birth should alert physicians to the risk of poor cerebral hemodynamic status. In our illustrated case, MCA pseudo-feeders were clearly visible on the MRI performed at birth (indicated by arrows, Figures 1A–C). It is evident that more data is required to draw any reliable conclusions. However, in future similar cases, we will consider emergency embolization in a tertiary center that is well-versed in cerebral endovascular treatment in neonatal patients, targeted to the largest shunts in order to improve cerebral and cardiac hemodynamics to prevent permanent brain damage.

Nowadays, historical neonatal scores that do not take into consideration cerebral MRI findings, especially the Bicetre neonatal evaluation score, are no longer appropriate in clinical practice. Early identification of relevant prognostic factors should help to improve the challenging management of fetuses and newborns with VGAM.
