# Erratum: Pseudo-feeders as a red flag of impending or ongoing severe brain damage in Vein of Galen Aneurysmal Malformation

**DOI:** 10.3389/fped.2023.1170918

**Published:** 2023-03-03

**Authors:** Frontiers Production Office

**Affiliations:** Frontiers Media SA, Lausanne, Switzerland

**Keywords:** Vein of Galen Aneurysmal Malformation, outcome, prognostic factor, encephalomalacia, pseudo-feeders

An Erratum on Pseudo-feeders as a red flag for impending or ongoing severe brain damage in Vein of Galen aneurysmal malformation By Saliou G and Buratti S. (2022) Front. Pediatr. 10:1066114. doi: 10.3389/fped.2022.1066114

An omission to the funding section of the original article was made in error. The following sentence has been added: “Open access funding was provided by the University of Lausanne”

The original version of this article has been updated.

